# Mothers' education and ANC visit improved exclusive breastfeeding in Dabat Health and Demographic Surveillance System Site, northwest Ethiopia

**DOI:** 10.1371/journal.pone.0179056

**Published:** 2017-06-28

**Authors:** Amare Tariku, Kassahun Alemu, Zemichael Gizaw, Kindie Fentahun Muchie, Terefe Derso, Solomon Mekonnen Abebe, Mezgebu Yitayal, Abel Fekadu, Tadesse Awoke Ayele, Geta Asrade Alemayehu, Adino Tesfahun Tsegaye, Alemayehu Shimeka, Gashaw Andargie Biks

**Affiliations:** 1 Department of Human Nutrition, Institute of Public Health, College of Medicine and Health Sciences, Institute of Public Health, University of Gondar, Gondar, Ethiopia; 2 Dabat Research Center Health and Demographic Surveillance System, Institute of Public Health, College of Medicine and Health sciences, University of Gondar, Ethiopia; 3 Department of Epidemiology and Biostatistics, Institute of Public Health, College of Medicine and Health sciences, University of Gondar, Gondar, Ethiopia; 4 Department of Environmental and Occupational Health and Safety, Institute of Public Health, College of Medicine and Health sciences, University of Gondar, Ethiopia; 5 Department Health Service Management and Health Economics, Institute of Public Health, College of Medicine and Health sciences, University of Gondar, Ethiopia; TNO, NETHERLANDS

## Abstract

**Introduction:**

Despite its proven benefit in reducing child mortality and morbidity, the coverage of exclusive breastfeeding (EBF) remains sub-optimal. In Ethiopia, about 52% of infants under six months of age were exclusively breastfed, implying the need for further identification of the barriers to optimal EBF practice. Therefore, this study aimed to investigate EBF and its determinants in the predominantly rural northwest Ethiopia.

**Methods:**

The study was conducted at the Dabat Health and Demographic Surveillance System (HDSS) site, which is located in Dabat District, northwest Ethiopia. A total of 5,227 mothers with children under five years of age were included for analysis. Multivariable binary logistic regression analysis was employed to identify factors associated with EBF. The Adjusted Odds Ratio (AOR) with a 95% Confidence Interval (CI) was estimated to show the strength of association. A P-value of <0.05 was used to declare statistical significance.

**Results:**

About 54.5% [95% CI: 51.9, 57.1] of the mothers practiced EBF. Mothers' education [AOR = 2.10; 95% CI: 1.63, 2.71], age (20–35 years) [AOR = 1.39; CI: 1.07, 1.80], urban residence [AOR = 1.28; 95% CI: 1.07, 1.54], at least one ANC visit [AOR = 1.41; 95% CI: 1.23,1.61], initiation of breastfeeding within one hour of birth [AOR = 1.32; 95% CI: 1.15,1.50], richer household [AOR = 1.34; 95% CI: 1.07, 1.65], and withholding prelacteal feeds [AOR = 1.34; 95% CI: 1.17, 1.53] were found important determinants of EBF.

**Conclusion:**

In this study area, the prevalence of EBF is lower than the national as well as the global recommendation for universal coverage of EBF. Therefore, strengthening the implementation of Infant and Young Child Feeding strategy (IYCF) and maternal health care utilization are essential for stepping up EBF coverage. Moreover, attention should be given to uneducated, rural resident, and adolescent mothers.

## Introduction

Exclusive breastfeeding (EBF) is an ideal way of achieving optimal child growth and development [1,2]. Because of an adequate amount of omega-3 poly-unsaturated fatty acids and other bioactive ingredients in the mother's milk, EBF improves the brain development of the child, its intelligence and the capacity to learn [3–6]. Globally, EBF is noted as a major child survival strategy [1,2], provided that the implementation of optimal EBF is guaranteed with a significant reduction in the risk of morbidity and mortality for a decade [1,7,8]. Decreased odds of acquisition of infectious diseases, such as pneumonia [8], diarrhea [7, 9] and upper respiratory tract infection [10,11] are particularly documented among exclusively breastfed children. Besides, a prolonged duration of EBF is protective against the risk of non-communicable diseases, like asthma and other allergic related respiratory infections [12,13], celiac diseases [14,15], atopic dermatitis [13], and diabetes mellitus [16]. Furthermore, it provides emotional and psychological benefits by stepping-up the mother-child bonding [17].

Despite its proved benefits, the global EBF practice remains sub-optimal, resulting in only 39% of the infants in developing countries being exclusively breastfed for the first six months. In southern Asia, EBF practice is estimated to be at 45% [18], and only 24.2% of infants are exclusively breastfed in Brazil [19]. On the other hand, the lowest prevalence (16%) is documented in the United States of America [20]. In Africa, modest improvement in the prevalence of EBF (22% to 35%) is observed in the last fifteen years, still it is lower than the global average [18]. The lowest rate of EBF is noted in some African countries, like Nigeria (16.4%) and South Africa (13.7%) [21,22]. In Ethiopia, breastfeeding is one of the culturally accepted nutritional practices; however, the rate of EBF (52%) [23] is by far lower than the universally accepted coverage, 90% [24]. Furthermore, it has declined to 32% at 4–5 months of age of infants [23].

Globally, a lower rate of EBF is explained mainly in relation to the level of maternal health care utilization and socio-demographic conditions. Accordingly, the higher odds of non-EBF are observed among unmarried [25–27], uni-parous [28–31], outdoor worker [31–34], poorly educated [28,34–36], younger (<20 years) [20], smoker, and drinker [27,30] mothers. Moreover, mothers from poorer households [21,25], have either no or incomplete antenatal care visits [21,29,32,36–38], and practice late initiation into breastfeeding [36,37] are experienced increased likelihood of early discontinuation of EBF. Different reports showed a controversial effect of residence on the duration of EBF. For instance, a study in Timor-Leste noted early cessation of EBF in urban settlements [33], whereas in Ethiopia, it is in the rural areas [31].

More than 10 million child deaths occur in the world each year from preventable causes, like diarrhea, pneumonia, measles, and undernutrition, the majority of which happen in developing countries [24]. In fact, promotion of optimal breastfeeding is confirmed to avert the preceding causes of death [1,5,7–11,24], particularly scaling-up EBF to 90% coverage is deemed to prevent 13% of all causes of child mortality [24]. Similarly, there is a high rate of child mortality in Ethiopia (88 per 1,000 live births), of which a substantial proportion occurs in rural areas, where a higher prevalence of sub-optimal breastfeeding is documented [23]. This problem suggests the need to further identify the barriers to optimal EBF practice. Therefore, this study aimed to investigate EBF and its determinants in the predominantly rural northwest Ethiopia.

## Methods

### Study setting

Dabat HDSS site is located in Dabat District, northwest Ethiopia. The site was established in 1996 and currently covers a total of 13 kebeles (9 rural and 4 urban kebeles, *smallest administration units in Ethiopia*) with 17,000 households and 69,468 inhabitants. The kebeles under the surveillance site were selected by taking all ecological zones (high land, middle land, and low land) into account. Dabat HDSS is a full member of the International Network of Demographic Evaluation of Populations and Their Health (INDEPTH), a network of 44 HDSSs from the Global South.

### Data collection and study population

Since its establishment, the Dabat HDSS site has been collecting information on vital events, such as birth, death, migration, pregnancy observation, marital status, and housing conditions every six months and verbal autopsy (VA) as the event happened. Re-census was planned to be done every 5 to 7 years. Thus, the latest was carried out from October to December2014 for a reconciliation purpose. A structured and pre-tested questionnaire was used to collect the data. The English version questionnaire was adapted and translated into the Amharic, the national language. A total of 30 data collectors, 13 field supervisors, and 50 local guiders were recruited and involved in the data collection processes. All of the filed assistants (data collectors and supervisors) were permanent employees of the HDSS. A five-day intensive training was given to data collectors and supervisors. The training focused on objectives, ethical issues, and techniques of interview. The questionnaire was piloted outside the study area. Clarity of questions, applicability of tools, and procedures were evaluated by the pretest.

All mothers with children aged less than 59 months and lived in the thirteen kebeles of the HDSS site were included in the study. According to WHO criteria, EBF was defined as the practice of feeding only breast milk (including expressed breast milk) during the first six months and no other liquids and solid foods except medications [2]. Accordingly, the duration of EBF was ascertained by asking the mothers a key question, “When did you first introduce any solid, semi-solid, or fluid to [child's name] in addition to breast milk''. The data collectors made mothers aware that providing medicines/syrups/supplements, except water, is not considered as giving additional food to the infant. However, prelacteal feeding is widely practiced in Ethiopia, including the Dabat District, study area [23,39]. In this case, using lifelong indicators might underestimate the EBF coverage as a result we excluded anything (prelacteal feeding) given to the infant in the first 2–3 days of life while measuring EBF [40].

To minimize recall bias, data collectors used different probing mechanisms, such as relating the time of initiation to known public events, occurrences of common childhood developmental milestones, and immunization schedules. Also, in case of mothers with more than one under five children, they were requested to respond about the youngest child.

### Data analysis

Data were entered into the Household Registration System (HRS) version 2.1 which was customized from other DHSS sites in Africa. The extracted data were exported to STATA version 14 for further analysis which also helps to automatically eliminate multi-collinear variables. The binary logistic regression model was employed to identify the determinants of EBF. Variables in the bivariable analysis with a P-value of <0.2 were entered into the multivariable logistic regression analysis to control confounders. Adjusted odds ratio (AOR) with the corresponding 95% confidence interval (CI) were estimated to show the strength of association. A P-value of <0.05 was used to declare statistical significance. Model fitness was checked using the Hosmer and Lemeshow goodness of fit test, and it was found at 0.72.

The household wealth index was computed for urban and rural residents separately, using the principal component analysis. In the final model, the common factor scores were summed and ranked into lowest, middle, second, fourth, and highest.

### Ethics approval and consent to participate

The study protocol was ethically approved by the Ethical Review Board (IRB) of the University of Gondar. Written informed consent was obtained from the head of the household. The study posed a low or not more than a minimal risk to the study participants. Also, the study did not involve any invasive procedures and reporting of any response for intervention. Accordingly, after the objective of the study was explained, verbal consent was secured from the respondents of the study. Moreover, the confidentiality of information was guaranteed by using code numbers rather than personal identifiers and by keeping the data locked.

## Result

A total of 5,227 mothers with children aged under five years were included in the study. Two-third (65.3%) of the mothers were aged 20–34 years. Of all mothers, 3313 (63.4%) were illiterate, while only 729 (13.9%) attended secondary school and above. A total of 3940 (75.4%) mothers were rural inhabitants, and 4481 (85.7%) were married at moment (Table 1). Among the total, 3332 (67.7%) of the mothers had at least one ANC visit for the index child and 3135 (63.7%) of them were not currently using any contraceptive methods. Almost all, 5,175 (99.01%) of the mothers breastfed their index children at least once in the child's life. Furthermore, early initiation of breastfeeding was reported by 2282 (44.1%) mothers, and prelacteal feeding by 3,054 (58.4%).

**Table 1:** Socio-demographic characteristics of the mothers in Dabat HDSS site, northwest Ethiopia, October to December 2014.

Variables	Frequency	Percentage
**Mother's age**		
15–19	303	5.8
20–35	3416	65.3
36–54	1508	28.9
**Mother's education**		
Illiterate	3313	63.4
First cycle primary school	723	13.9
Second cycle primary school	462	8.8
Secondary school and above	729	13.9
**Mother's marital status**		
Currently married	4481	85.7
Currently unmarried*	746	14.3
**Mother's religion**		
Christian	5057	96.7
Muslim	170	3.3
**Mother's ethnicity**		
Amhara	5,222	99.9
Other	5	0.1
**Place of residence**		
Rural	3940	75.4
Urban	1287	24.6
**Wealth status (5072)**		
Poorest	665	13.1
Second quintile	924	18.2
Middle quintile	1174	23.1
Fourth quintile	1133	22.3
Highest quintile	1176	23.3
**Age at first marriage (4921)**		
<18	4,135	84.0
≥18	786	16.0

*single, divorced, and widowed.

In this study area, about 54.5% [95% CI: 51.9, 57.1] of mothers exclusively breastfeed their children (Table 2). In addition, the median duration of EBF was 6 months (the inter-quartile range lies between 6 and 8 months).

**Table 2:** Health related characteristics of the mothers in Dabat HDSS site, northwest Ethiopia, October to December 2014.

Variables	Frequency	Percentage
**At least one ANC visit (4921)**		
Yes	3332	67.7
No	1589	32.3
**Breastfeeding**		
Yes	5,175	99.0
Never breastfed	52	1.0
**Initiation of BF (5175)**		
Early initiation^&^	2282	44.1
Late initiation^&&^	2893	55.9
**Prelacteal feeding**		
No	2,173	41.6
yes	3,054	58.4
**Non communicable disease**^$^		
Yes	110	2.1
No	5117	97.9
**Maternal alcohol consumption (5173)**		
Never	323	6.2
Sometimes	4,850	93.8
**Cigarette smoking (5173)**		
Never	5153	99.6
Sometimes	20	0.4
**Energy demanding work (5173)**		
Not energy demanding	230	4.4
Somehow	3300	63.8
Highly energy demanding	1643	31.8
**Current use of contraceptive methods (4921)**		
Yes	1786	36.3
No	3135	63.7
**Past use of contraceptive methods**		
No	2393	45.8
Short acting**	2666	51.0
Long acting***	168	3.2

**Depo-Provera and oral contraceptive pills

***nor-plant and Intra uterine contraceptive device

^&^ Initiation of breastfeeding within 1h of delivery

^&&^ Initiation of breastfeeding after 1h of delivery

$ affirmed by asking mothers whether they had established chronic disease or not, such as hypertension, diabetes mellitus, cancer, heart problem, and asthma

### Determinants of exclusive breastfeeding

In the multivariable analysis, mother's age, residence, educational status, ANC visit, household wealth status, initiation of breastfeeding and prelacteal feeding remained significant determinants of EBF.

Accordingly, the odds of EBF were high among mothers aged 20–35 years [AOR = 1.39; CI: 1.07, 1.80], and lived in the urban kebeles [AOR = 1.28; 95% CI: 1.07, 1.54] of the study area. Compared to illiterate mothers, the likelihood of EBF increased among mothers who attended first cycle primary school [AOR = 1.30; CI: 1.09, 1.55], second cycle primary school [AOR = 1.50; 95% CI: 1.19, 1.87], and secondary school and above [AOR = 2.10; 95% CI: 1.63, 2.71]. Though marital status showed a marginal significance, the odds of EBF were higher among married women [AOR = 1.20; 95% CI: 0.99, 1.46] compared to their unmarried counterparts.

Mothers who had at least one ANC visit [AOR = 1.41; 95% CI: 1.23,1.61] and initiated breastfeeding early [AOR = 1.32; 95% CI: 1.15,1.50] experienced higher odds of EBF. Also, mothers who did not give prelacteal feeds to their children had increased odds of EBF [AOR = 1.34; 95% CI: 1.17, 1.53]. The likelihood of EBF increases with improvements in household wealth status. Accordingly, the more odds of EBF were noted among households found in the highest [AOR = 1.34; 95% CI: 1.07, 1.65] wealth category (Table 3).

**Table 3:** Determinants of EBF among mothers in Dabat HDSS site, northwest Ethiopia, October to December 2014.

Variables	Exclusive breastfeeding			
	Yes	No	COR (95% CI)	AOR (95% CI)
**Mother's age**				
15–19	148	155	1	1
20–35	1,942	1,474	1.38 (1.09, 1.75)	1.39 (1.07, 1.80)*
36–54	761	747	1.07 (0.83, 1.37)	1.25 (0.94, 1.65)
**Educational status**				
Illiterate	1,609	1,704	1	1
First cycle	407	316	1.36 (0.83, 1.37)	1.30(1.09, 1.55)*
Secondary cycle	282	180	1.66 (1.36, 2.02)	1.50 (1.19, 1.87)*
Secondary and above	553	176	3.33 (2.77, 3.99)	2.10(1.63, 2.71)*
**Marital status**				
Married	2,428	2,053	0.90 (0.77, 1.06)	1.20 (0.99, 1.46)
Unmarried	423	323	1	1
**Religion**				
Christian	2,732	2,325	1	1
Muslim	119	51	1.99 (1.43, 2.77)	1.84 (0.92, 3.01)
**Residence**				
Rural	1,978	1,962	1	1
Urban	873	414	2.09 (1.83, 2.39	1.28 (1.07, 1.54)*
**Wealth index (5072)**				
Poorest	329	336	1	1
second quintile	464	460	1.03 (0.84, 1.26)	1.13 (0.91, 1.40)
Middle quintile	646	528	1.25 (1.03, 1.51)	1.21 (0.98, 1.49)
Fourth quintile	639	494	1.32 (1.09, 1.60)	1.34 (1.08, 1.65)*
Richest quintile	678	498	1.39 (1.15, 1.68)	1.34 (1.07, 1.65)*
**Age at first marriage (4921)**				
<18	2,169	1,966	1	1
≥18	523	263	1.80 (1.54, 2.12)	1.03 (0.84, 1.25)
**At least one ANC visit for the index child (4921)**				
Yes	1,979	1,353	1.79 (1.59, 2.03)	1.41(1.23,1.61)*
No	713	876	1	1
**Prelacteal feeding**				
No	1,375	798	1.84 (1.67, 2.06)	1.34 (1.17, 1.53)*
Yes	1,476	1,578	1	1
**Initiation of breastfeeding (5175)**				
Late initiation	1,423	1,470	1	1
early initiation	1,401	881	1.64 (1.47, 1.84)	1.32 (1.15,1.50)*
**Alcohol consumption (5173)**				
Never	205	118	1.49 (1.18, 1.88)	1.09 (0.78, 1.54)
Sometimes	2,614	2,236	1	1
**Energy demand for main job (5173)**				
Not energy	154	76	1.76 (1.31, 2.35)	1.28 (0.92,1.79)
Somehow demanding	1,785	1,515	1.02 (0.91, 1.15)	0.99 (0.87,1.12)
Highly demanding	880	763	1	1

*significant at a P-value of<0.05

## Discussion

This study demonstrated that the proportion of mothers who exclusively breastfeed their infants for six months was lower than the national as well as the global recommendation for a universal coverage of EBF. Mothers' age, residence, educational status, ANC visit, initiation of breastfeeding, prelacteal feeding, and household wealth status were the key determinants of EBF.

In this study, the prevalence of EBF was consistent with the 2011 Ethiopian Demographic and Health Survey (DHS) report (52%) [23], and that of other local studies, like the districts of Ankesha Guagusa (53%) [31] and Bahir Dar (50.3%) [41] in northwest Ethiopia. The current prevalence of EBF showed an improvement over that of a previous longitudinal study conducted in the same study area [32]. This progress was supported by the national EBF coverage improvement shown by the two Ethiopian DHS reports, 49% in 2005 and 52% in 2011 [23,42]. The proportion was greater than that of recent estimate of the prevalence of EBF in developing countries (39%) [18], and such other African countries as Nigeria (16.4%) [21] and Egypt (9.7%)[37].

The improved coverage of EBF in this study area could be attributed to the current intensified efforts of the government and other partners (UNICEF) to promoting appropriate IYCF practice, like Health Extension Program and the IYCF strategy [43]. Compared to this study, a lower rate of EBF is also reported in developed countries, such as Canada (15.3%) [28] and the United States of America (USA) (16.8%) [20]. This could be related to better economic status of mothers in developed countries which in turn stepped-up the affordability and accessibility of formula milk. Also, unfavorable attitude of mothers toward EBF coupled with the aggressive promotions of formula milk might have influenced them to choose breast milk substitutes [28,44]. In addition, poor maternal (pregnancy and postnatal) leave systems might explain the low coverage of EBF in western countries.

However, the prevalence of EBF in this study was lower than the national target of 70% set for 2015 [45] and the global recommendation of 90% for universal coverage [24]. Therefore, this evidence suggests the need for further intensification of current efforts [43].

Higher odds of EBF were noted among educated mothers compared to those of uneducated counterparts. The finding was in agreement with reports elsewhere [28,34–36]. Maternal education has a valuable role in improving child breastfeeding and caring practices [46–48]. Moreover, better maternal health care utilization (ANC and institutional delivery), which is the most popular strategy for improving EBF coverage, is documented among educated women [21, 23, 29,32,35–37].

The odds of EBF were 39% higher among mothers aged 20–35 years compared to those who were aged 15–19 years. A similar finding was reported in the United States of America [20]. In fact, mothers in their adolescence were poorly empowered and were less likely to make decisions on child health and caring practices [23]. In addition, in developing countries, including Ethiopia, the majority of adolescent mothers had limited nutrition and child feeding knowledge [49,50].

The study has shown that the odds of EBF were better among mothers who had at least one ANC visit for the index child as compared to their counter parts. This report was consistent with previous study findings elsewhere [21,29,32,36,37]. In fact, ANC visit is an entry point to improve mothers' breastfeeding behavior by providing nutrition education and counseling [1,32]. It is also evident that prenatal breastfeeding counseling significantly improves EBF practice [38,51].

Like previous research findings [25–27], our study indicated that the odds of EBF were higher among married women compared to their unmarried counterparts. This could be explained by the roles of husband in enhancing women's confidence to practice appropriate breastfeeding [52]. Paternal favorable attitude and involvement in breastfeeding counseling was significantly associated with optimal duration of EBF [52–54]. Partner involvement has a special importance for Ethiopian women most of whom are less empowered in making household related decisions [23].

In this study, not giving prelacteal feeds was associated with the higher odds of EBF. The report was supported by previous studies, in which prelacteal feeding was negatively associated with breastfeeding practices, such as non-exclusive breastfeeding, late initiation of breastfeeding, and early cessation of same [55–57]. It mainly operates through impairing infants frequency of suckling [23]. On the other hand, this study identified early initiation of breastfeeding as an independent predictor of EBF was consistent with reports elsewhere [36,37]. This is likely to be related to current improvements in the unitization of institutional delivery in the study area. Inappropriate neonatal feeding practices (prelacteal feeding and late initiation of breastfeeding) were not common among mothers who gave birth at health facilities [23,39,57].

Our study noted higher odds of EBF among mothers who lived in urban kebeles, and the finding was in line with another local report [31]. This could be attributed to the disparities in mothers' health care access, utilization and health seeking behavior which is by far higher in urban areas of Ethiopia [23]. On the other hand, health care utilization and health seeking behavior also varied based on household wealth status [23]. Likewise, such type of social inequalities could explain our result indicating the positive effect of household wealth status on EBF, according to which the likelihood of EBF increased with improvements in household wealth status. The finding is supported by another report from Ethiopia [25]and Nigeria [21].

The study has attempted to show EBF rate and its determinants in a well-defined population (HDSS site) representing northwest Ethiopia. The study also involves an adequate sample which ultimately improved the power of the study.

However, the study had limitations. Firstly, during data cleaning, some of the variables were found with missing value; thus efforts were made to manage the missing values by tracing and cross-checking the questionnaire, maintaining the identification number. However, some of the values continued to be missing in the hard copy. Given that, the frequency within each explanatory variable is not necessarily equal.

Secondly, the study did not collect data on the frequency of ANC visits. Thus, as the measurement of variables depended on the recall of respondents, there was a possibility of committing social desirability and recall bias in ascertaining EBF with a maximum recall period of 59 months. But, strong efforts were made to minimize the limitations mainly through recruiting experienced data collectors and supervisors who had been working in the HDSS site for a decade or more. Intensive training regarding the techniques of interview was given to field assistants, and close supervisions were also made. Furthermore, in almost all of the cases, mothers were requested to respond on the children’s' feeding practices.

## Conclusion

The prevalence of EBF in the predominantly rural northwest Ethiopia was lower than the national as well as the global recommendation for the universal coverage of EBF. EBF was significantly associated with socio-demographic and health care utilization related characteristics. Moreover, inappropriate neonatal feeding practices were correlated with non-EBF practice. Therefore, strengthening the implementation of the IYCF strategy and maternal health care utilization is essential to increase EBF coverage. Moreover, interventions aiming to promote IYCF knowledge and practice of mothers should pay attention to the uneducated, rural resident and adolescent mothers.
